# Rapid Identification of Buprenorphine in Patient Saliva

**DOI:** 10.4172/2155-9872.1000368

**Published:** 2017-06-23

**Authors:** Stuart Farquharson, Kathryn Dana, Chetan Shende, Zachary Gladding, Jenelle Newcomb, Jessica Dascher, Ismene L Petrakis, Albert J Arias

**Affiliations:** 1 Real-Time Analyzers, Inc., 362 Industrial Park Road, Unit 8, Middletown, CT 06457, USA; 2 Veteran Affairs CT Healthcare System, USA; 3 Yale University School of Medicine, USA

**Keywords:** Saliva analysis, Drug detection, Buprenorphine, Point-of-care, SERS, Veterans

## Abstract

Buprenorphine is becoming the medication of choice to help patients withdraw from opioid addiction. However, treatment is compromised by the inability of physicians to assess patient usage during scheduled examinations. Here we describe the development of a point-of-care (POC) analyzer that can rapidly measure both illicit and treatment drugs in patient saliva, ideally in the physician’s office, and with a degree of accuracy similar to chromatography. The analyzer employs a relatively simple supported liquid extraction to isolate the drugs from the saliva and surface-enhanced Raman spectroscopy (SERS) to detect the drugs. The SERS-based POC analyzer was used to identify buprenorphine and opioids in saliva samples by matching library spectra to samples collected from 7 veterans. The total analysis time, including sample preparation, was ~25 minutes. Buprenorphine concentration was estimated between 0 and 3 μg/mL. While no other prescription opioids were detected in any samples, heroin was identified in one sample; Δ-9 tetrahydrocannabinol (THC) was detected in 3 samples; and acetaminophen, caffeine, and nicotine were detected in several samples, none of which interfered with the measurements. The analysis was in very good agreement with urinalysis, correctly identifying the presence or absence of buprenorphine and THC in 13 of 14 measurements.

## Introduction

Since Operation Iraqi Freedom and Operation Enduring Freedom, there has been a significant increase in the use of opioids, such as OxyContin and Vicodin, by United States military personnel. In 2014, the Department of Veterans Affairs Office of the Inspector General (DVAOIG) reported just over 442,000 veterans receiving opioid treatment. Over 90% of these patients were diagnosed with pain or mental health issues, such as post-traumatic stress disorder, and nearly 60% with both [1]. In 2015, ~68,000 of the veterans taking opioids were characterized as having substance-use disorders (SUDs) [2].

In an effort to reduce current and future SUD patients, Veterans Affairs (VA) hospitals expanded the use of drugs to reduce opioid dependence and side effects [3,4]. One of the most successful medications for opioid treatment is buprenorphine. It has 25–40 times the potency of morphine [5], and is considerably less addictive. In 2003 the FDA approved this drug for office treatment by physicians. However, opioid treatments are not effective if patients discontinue medications or give in to withdrawal symptoms and re-initiate drug use. This is not uncommon, since most patients are not hospitalized and often treated as outpatients. In effect, it is the patient’s responsibility to take treatment drugs according to the prescribed schedule. Consequently, the VA Clinical Practice Guideline calls for initial and frequent urine drug testing to identify discontinuation of medications or any recurrence of drug use, and adjust treatment appropriately. However, the 2014 DVAOIG report indicates that a very low percent of veterans take the follow-up urine tests [1]. This low percentage may be attributed to the clinical setting and methods used to analyze urine [1].

Currently, there are two types of analysis for monitoring patient compliance: immunoassay kits and liquid or gas chromatography coupled to mass spectrometers (LC- or GC-MS). Commercially available immunoassay kits, while portable and somewhat usable in outpatient settings, have several limitations. Specifically, they detect a limited set of drugs; they take as much as 1.5 h to perform; [6] they are prone to false positives (as high as 25% for buprenorphine [7]); and they only determine presence or absence of a drug above or below a predefined threshold. Conversely, LC- and GC-MS can measure virtually all drugs and are highly accurate and quantitative, but measurements take hours involving extensive sample preparation and instrument calibration, requiring skilled operators in a laboratory setting [6,8–10]. Furthermore, both devices use urine samples, which complicate analysis since the parent drugs are metabolized and typically are not excreted in urine until 1–3 days after initial use. Saliva sample analysis is available by some laboratories, but again is usually only available by sending out a sample from the clinic, with several days’ delay in receiving results. Finally, the authenticity of a urine sample may be compromised by intentional sample tampering [11].

Consequently, there remains a critical need for a point-of-care (POC) device that combines the portability of immunoassay kits with the identification and quantitation abilities of LC- or GC-MS so that health care personnel can assess SUD patient compliance in outpatient settings. Toward developing such a device, we have been investigating the potential of surface-enhanced Raman spectroscopy (SERS) to both identify and quantify drugs in saliva [12–14]. The expected success of this approach is based on the extreme sensitivity of SERS [15,16], the ability to measure very small samples, such as <mL of saliva, and the ability to identify molecular structures of drugs through the rich vibrational information provided by Raman spectroscopy. Furthermore, saliva represents an ideal sample medium, since collection is non-invasive, can be performed in the presence of health care personnel (eliminating the chance of sample tampering), and, most importantly, it has been shown that drug concentrations in saliva are typically equivalent to those in blood plasma. In the case of intravenous injection, buprenorphine concentrations in both saliva and blood plasma are typically in the 0.5 to 5 ng/mL range 1–2 h after administration [17,18]. However, saliva concentrations after sublingual administration can remain as high as 500 ng/mL per mg of dose after 5 h [17]. Here we present the initial development of a SERS-based POC device and its use to detect buprenorphine extracted from saliva provided by veterans undergoing treatment.

## Materials and Methods

### Materials

All solvents and chemicals used to prepare samples, colloids, and perform extraction were obtained from Sigma-Aldrich (St Louis, MO). The drugs used to prepare the spectral library were prepared from 1 mg/mL methanol forensic samples obtained from the same supplier. The supported liquid extraction columns were obtained from Biotage (Charlotte, NC). Whatman 42 glass microfiber filters and glass support slides were obtained from VWR (Radnor, PA).

### Methods

A 200-μL saliva sample mixed with 200 μL of distilled water was added to a supported liquid extraction column attached to an in-house vacuum line. The sample was adsorbed onto the support by applying a negative pressure of 15 inches of Hg for 1 sec. After 5 min, two sequential aliquots of 900 μL dichloromethane were drawn through the support, first using gravity for 5 min, then using −15 inches Hg for 1 min. The collected sample was dried under a gentle stream of nitrogen, and then reconstituted using 100 μL of distilled water.

A gold colloid solution for SERS was synthesized following a modified Lee-Meisel method [19]. Briefly, 240 mg of gold chloride (HAuCl_4_•3H_2_O) was dissolved in 500 mL water and heated to 100°C, at which time 50 mL of 1% sodium citrate was added and allowed to boil for 1 h. 50 mL aliquots of the colloid solution at room temperature were sequentially centrifuged (6000–9000 rpm, 10–30 min) and the concentrate was collected to obtain a final concentration increased by a factor of 20–30 times.

20 μL of the reconstituted sample was then mixed with an equal volume of the gold colloid solution and deposited onto a glass microfiber filter attached to a glass slide, which was placed on an XY sample stage for SERS measurements. Each spectrum consisted of five 1–sec acquisitions collected at 5 spots spaced 1 mm apart along the surface of the slide and averaged using ~30 mW of 785 nm laser excitation using an in-house Raman spectrometer and collection software (RTA LabRaman and Vista). Spectral analysis was performed using an in-house software program (S-Quant) as described below.

The drugs used to prepare the spectral library were prepared from 1 mg/mL methanol forensic samples that were diluted to 100 μg/mL using distilled water. 20 μL aliquots of these diluted drug samples were mixed with 20 μL of the gold colloids and measured as described above. The same procedure was used to prepare a buprenorphine reference sample at 10 μg/mL. The drugs measured are listed in Table 1.

## Results and Discussion

VA patients being treated for SUDs that already were providing urine samples were recruited to provide saliva samples according to IRB Protocol 00008942 (Chesapeake IRB, Inc.), the Human Subjects Subcommittee of the VA Connecticut Healthcare System (West Haven, CT) and by the Yale IRB. The participants went through an informed consent process, stated that they understood the study, and signed the consent and HIPAA privacy documents. This report describes 7 VA subjects who were participating in a larger study. These 7 participants were recruited while undergoing buprenorphine treatment for opioid use at least 2 weeks prior to providing a saliva sample, except patient number 2, who had just begun the treatment. They also provided information regarding drug use for the previous 2 weeks. Buprenorphine was administered sublingually as Suboxone at a 2 or 8 mg buprenorphine dose with naloxone at 1/4^th^ the buprenorphine dose (Table 2).

The participants did not eat or drink for 10 min prior to sample collection, which was performed by either spitting into plastic tubes, buccal swabbing, or a combination of the two methods until ~1 mL of saliva was obtained. The samples were sealed and refrigerated until saliva analysis was performed. For some participants, urine samples were collected earlier the same day as part of their clinic visit or participation in other research studies. For the remaining participants, urine samples were collected at the time of saliva collection. All urine samples were analyzed by the VA Medical Center laboratory. Urine samples were analyzed for amphetamines, barbiturates, benzodiazepines, buprenorphine, cannabinoids, cocaine, methadone, opiates, and oxycodone. Urinalysis indicated that 6 of 7 samples contained the treatment drug, buprenorphine; Sample 2 tested negative. Samples 1, 3, and 4 also tested positive for cannabinoids, as a general indicator of cannabis use. No other drugs were detected.

**Figure 1:**
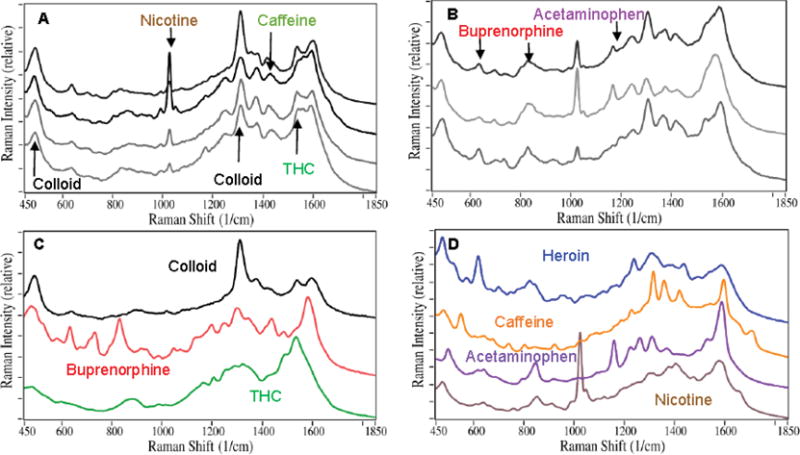
Stacked SERS of A) Samples 1–4 (top to bottom); B) Samples 5–7 (top to bottom); C) colloid, buprenorphine, and Δ-9 THC (top to bottom); and D) heroin, caffeine, acetaminophen, and nicotine (top to bottom). **Conditions:** ~1 mL saliva sample treated for analysis, ~50 mW of 785 nm laser excitation, 5 to 15 five-sec spectra averaged and smoothed (3^rd^ order polynomial, 11-point sliding smooth). The most unique peaks for each substance in the saliva sample spectra are indicated by arrows.

The 7 saliva samples were prepared according to the procedure described above, and their SERS were measured (Figure 1A and B). The initial SERS analysis was performed by fitting the measured spectra with weighted contributions from previously collected spectra for buprenorphine and Δ-9 tetrahydrocannabinol (THC), the principle psychoactive component of cannabis, and the background spectrum produced by the colloids. This approach indicated that all of the samples contained buprenorphine, and Samples 1, 3, and 4 contained THC. However, the weighted contribution of buprenorphine for Sample 2 and the contribution of THC for Sample 1 were both less than 1%. It also became apparent that additional substances were present (Figure 1C and D). Specifically, all of the samples contained nicotine, indicative of tobacco use (characterized by the narrow peak at 1030 cm^−1^), which is consistent with the fact that several patients indicated that they were smokers on their demographic forms. Samples 4, 5, and 6 contained acetaminophen (characterized by the peak at 1170 cm^−1^), while Samples 2 and 5 contained caffeine (characterized by peaks at 1430 and 1700 cm^−1^).

**Figure 2:**
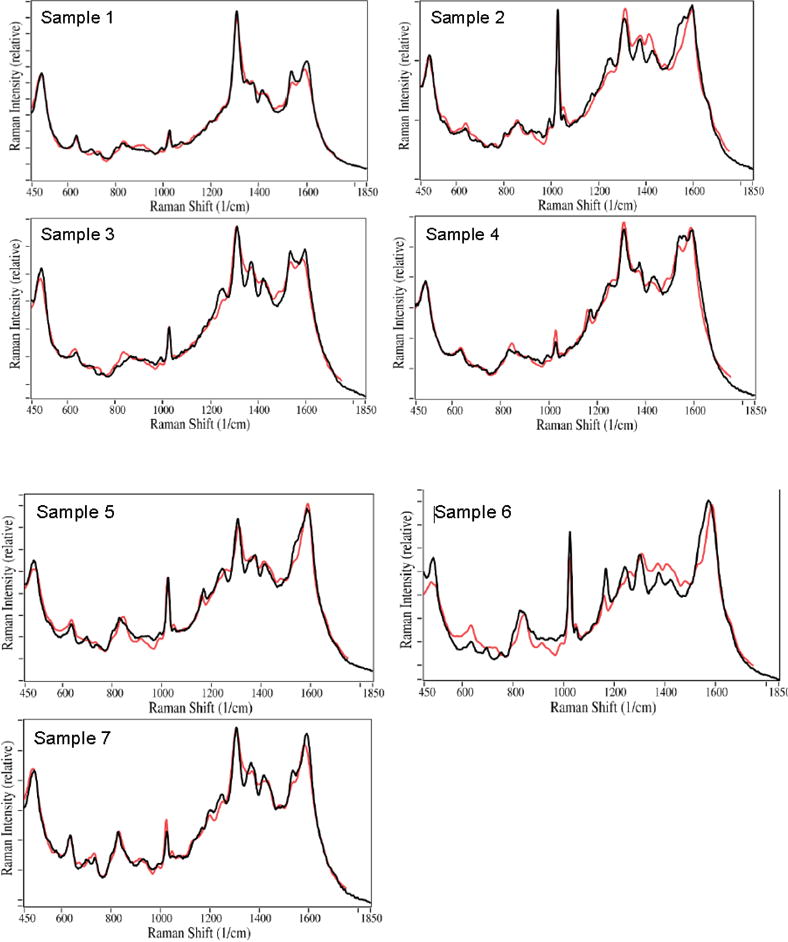
SERS of Samples 1–7. Actual spectra (black) are fit by the S-Quant software (red). All samples except Sample 6 include contribution from the colloid.

To better determine the drugs present in each sample, their spectra were fit using all of the spectra in a previously measured SERS library of 33 common over-the-counter, prescription, and illicit drugs. Relatively good fits were obtained after all contributions less than 1% were removed. In general, the fits consisted of acetaminophen, buprenorphine, caffeine, THC, and nicotine, as well as the colloid contribution (Figure 2). The spectral contributions total 100% for the fits presented. This procedure indicated that Sample 2 contained less than 1% buprenorphine (characterized by the peak at 835 cm^−1^), and therefore was removed in the final fitting process, but so was THC for Sample 1. This procedure was consistent with the urinalysis result for Sample 2 buprenorphine, but not Sample 1 THC. This procedure also indicated that Sample 3 contained heroin with a ~9% weighted contribution. While this was surprising, examination of the patient’s consent form indicated that heroin had been taken the day before the saliva was collected. The weighted spectral contributions for all of the samples are listed in Table 2. As indicated, all of the Samples, except Sample 6, required a significant colloid contribution to generate a reasonable fit. In fact, the above spectral fitting procedure indicated that less than 1% colloid contributed to the spectrum, and it was not included. The table also indicates drug contributions, totaling 100%, if the colloid contribution is removed. Note that the fits are not perfect, suggesting that other drugs, biochemical or saliva components, not in the library, are present in the sample.

An initial attempt was made to quantify the amount of buprenorphine by comparing its 835 cm^−1^ peak intensity to that for a single reference sample prepared at 10 μg/mL of buprenorphine spiked in drug-free saliva. The estimated concentrations for Samples 1, 4, 5, 6, and 7 ranged from 1.5 to 3.5 μg/mL, while Samples 2 and 3 were ~0.1 μg/mL. These values are consistent with studies that indicate the buprenorphine concentration is between 1 and 10 μg/mL in saliva for a single 1.0 mg dose between 5 and 20 h after sublingual administration [17]. However, these concentrations cannot be related to the patient doses, since a comprehensive calibration curve was not prepared at the time of the measurements, and the times between dose administration and saliva collection are unknown.

## Conclusion

This study demonstrated the potential of a SERS-based POC analyzer to detect drugs in patient saliva. Semi-automated spectral analysis, employing a spectral library, was able to identify and determine the relative contributions of the drugs present. This included the identification of heroin, acetaminophen and caffeine without prior knowledge of their presence in saliva samples. Furthermore, the presence of these additional drugs not only did not interfere with the measurements, but improved the analysis. Nevertheless, the method could be improved by using a library tailored to the patient population, such that it is not excessive in size, but includes only those drugs that could be reasonably expected in a sample. Finally, the SERS analysis was in very good agreement with urinalysis, correctly identifying the presence or absence of buprenorphine and THC in 13 of 14 measurements. Future work will involve measuring samples from a much larger population, and improving the analyzer sensitivity, quantitation, speed, and ease-of-use.

## Figures and Tables

**Table 1:** List of 33 drugs included in the SERS library. All were measured at 100 μg/mL.

acetaminophen	cannabidiol	heroin	meperidine	naltrexone	phenylbarbitol
amobarbitol	cocaine	hydrocodone	mescaline	nicotine	secobarbitol
amphetamine	codeine	hydroxy-THC	methadone	oxazepam	varenicline
buprenorphine	Δ9-THC	ibuprofen	methamphetamine	oxycodone	
buproprion	diazepam	MDA	methylphenidate	oxymorphone	
caffeine	fentanyl	MDMA	morphine	phencyclidine	

**Table 2:** Sample information: daily buprenorphine dose, urinalysis and SERS analysis (percent contribution to each sample spectrum, values in parenthesis are percent’s excluding colloid).

Sample	1	2	3	4	5	6	7
**Dose (mg)/day**	6	0	2	12	24	4	8
**Urinalysis**							
Buprenorphine	Y	N	Y	Y	Y	Y	Y
Cannabinoids	Y	N	Y	Y	N	N	N
**SERS Analysis**							
Buprenorphine	10.0 (84.0)		2.9 (17.9)	8.5 (38.3)	17.2 (31.6)	(46.4)	27.7 (87.7)
THC	<1 (2.5)		1.5 (9.3)	4.1 (18.5)			
Nicotine	1.6 (13.5)	16.0 (100)	2.9 (17.9)	3.9 (17.6)	10.1 (18.6)	(32.1)	3.9 (12.3)
Acetaminophen				5.7 (25.7)	7.6 (13.9)	(21.5)	
Caffeine		25.2 (61.2)			19.5 (35.9)		
Heroin			8.9 (54.9)				
Colloid	88.2	58.8	81.4	77.8	45.6		68.4
